# Early onset of efficacy with erenumab for migraine prevention in Japanese patients: Analysis of two randomized, double‐blind, placebo‐controlled studies

**DOI:** 10.1002/brb3.2526

**Published:** 2022-02-24

**Authors:** Koichi Hirata, Takao Takeshima, Fumihiko Sakai, Noboru Imai, Yasuhiko Matsumori, Yoshihisa Tatsuoka, Yotaro Numachi, Ryuji Yoshida, Cheng Peng, Daniel D. Mikol, Gabriel Paiva da Silva Lima, Sunfa Cheng

**Affiliations:** ^1^ Department of Neurology Dokkyo Medical University Tochigi Japan; ^2^ Department of Neurology Tominaga Hospital Osaka Japan; ^3^ Saitama International Headache Center Saitama Japan; ^4^ Department of Neurology Japanese Red Cross Shizuoka Hospital Shizuoka Japan; ^5^ Sendai Headache and Neurology Clinic Sendai Japan; ^6^ Department of Neurology Tatsuoka Neurology Clinic Kyoto Japan; ^7^ Amgen K.K. Research & Development Tokyo Japan; ^8^ Amgen Inc. Global Biostatistical Science Thousand Oaks California USA; ^9^ Amgen Inc. Global Development Thousand Oaks California USA

**Keywords:** calcitonin gene‐related peptide, chronic migraine, episodic migraine, erenumab, onset of efficacy

## Abstract

**Purpose:**

In two 24‐week migraine prevention studies in Japan, erenumab was associated with significantly greater reductions in migraine frequency versus placebo over Weeks 13–24 (primary endpoint). This post hoc analysis evaluated the onset of efficacy within the first 4 weeks after the initiation of erenumab from the 24‐week double‐blind periods of these studies.

**Methods:**

Placebo‐adjusted differences in least squares mean (LSM) change from baseline in weekly migraine days (WMD) were assessed weekly in each study and by migraine type (episodic (EM]/chronic [CM]) (Study 20170609).

**Results:**

A total of 407 patients from Study 20120309 (70 mg: N = 135; 140 mg: N = 136; placebo: N = 136) and 261 patients from Study 20170609 ([EM] 70 mg: N = 78; placebo: N = 81; [CM] 70 mg: N = 52; placebo: N = 50) were included. For Study 20120309, onset of efficacy was observed as early as Week 1 in favor of erenumab versus placebo. Placebo‐adjusted differences in LSM (95% confidence interval [CI]) change from baseline in WMD at Week 1 were −0.38 (−0.71 to −0.05; *p *= .022) and −0.49 (−0.82 to −0.16; *p* = .004) in favor of erenumab 70 and 140 mg, respectively. For Study 20170609, significant placebo‐adjusted differences were observed with erenumab 70 mg at Week 1 in patients with EM (LSM [95% CI]: −0.55 [−0.97 to −0.12; *p* = .012]), and at Week 2 in patients with CM (LSM [95% CI]: −0.81 [−1.53 to −0.09; *p* = .028]) and for the overall population (LSM [95% CI]: −0.71 [−1.09 to −0.33; *p* < .001]).

**Conclusions:**

Erenumab treatment significantly reduced WMD compared with placebo. Onset of erenumab efficacy occurred as early as Week 1 in patients with migraine.

## 1 INTRODUCTION

Several oral preventive treatments are available for the prevention of migraine in Japan, including beta‐blockers, antidepressants, calcium channel blockers, and anticonvulsant drugs. Despite this, preventive treatment use remains low in Japan, with acute‐phase therapies commonly used for migraine treatment (Ueda et al., 2019). Reasons for low adherence and early discontinuation include a perceived lack of efficacy, poor tolerability, and delayed onset of efficacy (Meyers et al., 2019; Tassorelli et al., 2018; Ueda et al., 2019), with >60% of patients discontinuing treatment within the first 2 months of treatment (Meyers et al., 2019). However, reliance on acute‐phase therapies as a standalone treatment modality for migraine is not without clinical consequences, and may be associated with the development of overuse headache and transformation to chronic migraine (Torres‐Ferrús et al., 2020).

Monoclonal antibodies that target the calcitonin‐gene related peptide (CGRP) and its receptor have more recently been developed (Aimovig [prescribing information], 2018; Detke et al., 2020; Dodick et al., 2018), which target the underlying pathophysiology of migraine. These agents have been shown to possess both a rapid onset of efficacy and a favorable safety profile (Aimovig [prescribing information], 2018; Silberstein et al., 2020; Vu et al., 2017; Winner et al., 2019), and are therefore expected to improve adherence and long‐term patient outcomes. Erenumab, a monoclonal antibody against the CGRP receptor, is approved for the prevention of episodic migraine (EM) and chronic migraine (CM) in over 70 countries (Pharmaceutical and Medical Devices, 2021), including the United States and countries within the European Union based on positive results from global phase 2 and 3 trials (Dodick et al., 2018; Goadsby et al., 2017; Tepper et al., 2017). Two randomized, double‐blind, placebo‐controlled studies of erenumab for migraine prevention have subsequently been conducted in Japan (Sakai et al., 2019; Takeshima et al., 2021). In these studies, erenumab was associated with significantly greater reductions from baseline in mean monthly migraine days (MMD) versus placebo over Weeks 13–24 (primary endpoint). However, reductions were also observed as early as 4 weeks (the earliest prespecified time point at which efficacy was assessed). Based on these findings, a post hoc analysis was conducted to evaluate the time to onset of efficacy within the first 4 weeks after the initiation of erenumab in patients with EM and CM from the 24‐week double‐blind treatment periods of these studies in Japan.

## 2 METHODS

### 2.1 Study designs

Detailed information regarding study designs, populations, and results have been published previously (Sakai et al., 2019; Takeshima et al., 2021). Study 20120309 (NCT02630459) was a randomized, double‐blind, placebo‐controlled, pivotal phase 2 study of erenumab in adult patients (N = 475) with EM in Japan. Patients were eligible for inclusion if they had a history of migraine for ≥12 months, an average of ≥4 to <15 migraine days during the baseline period, and demonstrated ≥80% compliance with their electronic diary (eDiary). Patients were excluded if they were receiving ≥2 concomitant migraine preventive treatments, or had no therapeutic response (defined as no improvement in migraine frequency, severity, or duration after ≥6 weeks of treatment at the appropriate dose) to ≥3 migraine medication categories. Patients were randomized 2:1:2:2 to placebo or erenumab 28, 70, or 140 mg administered monthly by subcutaneous (SC) injection, with randomization stratified by migraine preventive treatment status (prior vs. current vs. none).

Study 20170609 (NCT03812224) was a randomized, double‐blind, placebo‐controlled, phase 3 pivotal study of erenumab in adult patients (N = 261) with EM and CM in Japan. Patients were eligible for inclusion if they had a history of migraine for ≥12 months, an average of <15 headache days with ≥4 migraine days (EM) or ≥15 headache days with ≥8 migraine days (CM) during the baseline period, and demonstrated ≥80% compliance with their eDiary. Patients were excluded if they had received ≥2 concomitant migraine preventive treatments or had no therapeutic response (defined as no improvement in migraine frequency, severity, or duration after ≥6 weeks of treatment at the appropriate dose) to ≥4 migraine medication categories. Patients were randomized 1:1 to placebo or erenumab 70 mg administered monthly by SC injection, with randomization stratified by migraine preventive treatment status (prior vs. current vs. none) and subtype (EM/CM).

**FIGURE 1:**
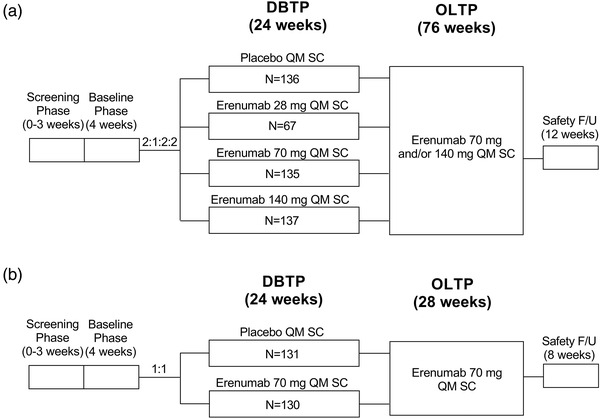
Study design: (a) Study 20120309 and (b) Study 20170609. F/U, follow‐up; QM, every month; SC, subcutaneous

Both studies consisted of a 24‐week double‐blind treatment period and an open‐label treatment period (Figure 1), in which patients received open‐label erenumab 70 or 140 mg QM SC (Study 20120309) or 70 mg QM SC (Study 20170609). Data for the 70‐ and 140‐mg dose groups from the 24‐week double‐blind period were used for this analysis.

Patients completed an eDiary daily with information about their migraine and nonmigraine headaches (including migraine/headache duration, most severe pain level, pain characteristics, and symptoms), patient reported outcomes (e.g., symptom interference and impact on physical functioning), and use of acute migraine therapies during the 4‐week baseline phase and subsequent double‐blind treatment phase. The primary endpoint in both studies was the change from baseline in MMD averaged over months 4, 5, and 6, with additional prespecified analyses at the end of each monthly treatment period. The primary objective of this post hoc analysis was the change from baseline in weekly migraine days (WMD) at Weeks 1─4 in the double‐blind treatment period of both studies. A migraine day was defined as per the International Classification of Headache Disorders 3rd edition (ICHD‐3) definition for migraine in both studies (Headache Classification Committee of the International Headache Society [HIS] 2013).

Studies were conducted in accordance with ethical principles of the Declaration of Helsinki, the International Council for Harmonisation (ICH) Harmonised Tripartite Guideline for Good Clinical Practice (GCP), and the Institutional Review Board (IRB) regulations. Clinical study protocols, informed consent forms, and other study‐related documents were reviewed and approved by the local or central IRBs of all study sites. Studies were conducted according to applicable local or regional regulatory requirements and in accordance with the responsibilities listed in the protocol. All patients or their legally accepted representative provided written informed consent to participate in each study. Both studies were registered at clinicaltrials.gov (NCT02630459 and NCT03812224).

### 2.2 Statistical analysis

Full details regarding statistical analysis methodology for each study have been published previously (Sakai et al., 2019; Takeshima et al., 2021). The full analysis set included all randomized patients and the efficacy analysis set included all patients who received ≥1 dose of study drug and had at least one change from baseline measurement in WMD available during Weeks 1−4. Post hoc analysis evaluated the change from baseline in WMD to assess the efficacy of erenumab versus placebo earlier than Week 4. The baseline WMD was calculated on the basis of the entire 4‐week baseline period (normalized into a 7‐d period). For change from baseline in WMD, the least squares mean (LSM) at each time point was calculated with adjusted analyses using a generalized linear mixed‐effects model that included treatment, visit, treatment‐by‐visit interaction, stratification factors, and baseline value as covariates and assumes a first‐order autoregression covariance structure, in each study and by migraine type (EM/CM) for the phase 3 study. Higher negative scores indicated greater reduction from baseline in the weekly average number of migraine days. No formal hypothesis testing was conducted for the endpoints included in this study and descriptive *p* values were reported without multiplicity adjustment. All statistical significances were considered nominal without further specification.

All statistical analyses were performed using SAS software version 9.4 (SAS Institute, Cary, NC, USA).

## 3 RESULTS

### 3.1 Patient disposition

A total of 408 patients received placebo (N = 136), erenumab 70 mg (N = 135), or erenumab 140 mg (N = 137) in Study 20120309 and 261 patients received placebo (N = 131) or erenumab 70 mg (N = 130) in Study 20170609, and comprised the full analysis set. Of these, one patient from the 140 mg group in Study 20120309 did not have a post‐baseline efficacy evaluation available and was excluded from the efficacy analysis set.

### 3.2 Patient characteristics

Baseline demographic and clinical characteristics of patients in Studies 20120309 and 20170609 have been published previously (Sakai et al., 2019; Takeshima et al., 2021) and were comparable with respect to age and gender. Mean (SD) WMDs were higher in Study 20170609 (placebo: 2.96 [1.43] days; 70 mg: 3.10 [1.50] days) than Study 20120309 (placebo: 1.92 [0.59] days; 70 mg: 1.96 [0.58] days; 140 mg: 2.04 [0.60] days) at baseline because ∼40% of patients in the former study had CM. A total of 9.3% and 34.6% of patients in Study 20120309, and 35.2% and 44.8% in Study 20170609 were currently receiving a migraine preventive treatment or had previously failed at least one migraine preventive treatment, respectively. Nearly all (>90%) patients in both studies were receiving acute migraine specific medication.

### 3.3 Change from baseline in weekly migraine days

**FIGURE 2:**
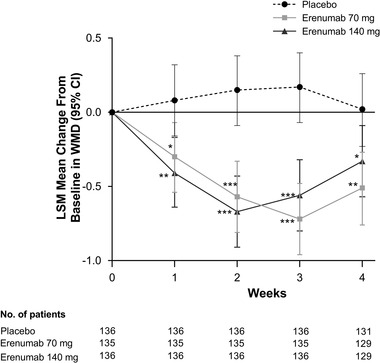
Change from baseline in weekly migraine days at Weeks 1–4 in Study 20120309. LSM, least squares mean. *p < .05; **p < .01; ***p < .001

In Study 20120309, reductions in WMD in favor of erenumab were observed as early as Week 1 and were sustained through Weeks 1−4 (Figure 2). LSM changes from baseline (95% CI) in WMD at Week 1 were 0.08 (−0.16 to 0.32) for placebo versus −0.30 (−0.54 to −0.07) for erenumab 70 mg and −0.41 (−0.64 to −0.17) for erenumab 140 mg. LSM changes from baseline (95% CI) at Week 4 were 0.02 (−0.23 to 0.26) for placebo versus −0.51 (−0.76 to −0.27) for erenumab 70 mg and −0.33 (−0.57 to −0.09) for erenumab 140 mg. Placebo‐adjusted differences in WMD in favor of erenumab versus placebo were significant at all time points evaluated for both the 70 and 140 mg dose. Placebo‐adjusted differences in LSM (95% CI) changes from baseline in WMD at Week 1 were −0.38 (−0.71 to −0.05; *p* = .022) for erenumab 70 mg and –0.49 (−0.82 to –0.16; *p* = .004) for erenumab 140 mg. Placebo‐adjusted differences in LSM (95% CI) changes from baseline in WMD at Week 4 were −0.53 (–0.86 to −0.19; *p* = .002) for erenumab 70 mg and −0.35 (−0.68 to −0.01; *p* = .042) for erenumab 140 mg.

**FIGURE 3:**
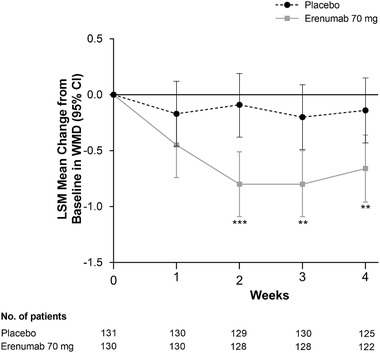
Change from baseline in weekly migraine days at Weeks 1–4 in Study 20170609. LSM, least squares mean. *p < .05; **p < .01; ***p < .001

In Study 20170609, numerical reductions in WMD were similarly observed as early as Week 1 with erenumab versus placebo (Figure 3), although the between‐treatment difference was not significant at this time point. LSM changes from baseline (95% CI) at Week 1 were −0.17 (–0.46 to 0.12) for placebo versus −0.45 (−0.74 to −0.16) for erenumab 70 mg. LSM changes from baseline (95% CI) at Week 4 were −0.14 (−0.43 to 0.15) for placebo versus −0.66 (−0.96 to −0.36) for erenumab 70 mg. Placebo‐adjusted differences in favor of erenumab versus placebo were observed at all time points in the overall population, which were significant at Weeks 2─4. Placebo‐adjusted differences in LSM (95% CI) change from baseline in WMD for erenumab 70 mg was −0.28 (−0.66 to 0.10; *p* = .15) at Week 1, −0.71 (−1.09 to −0.33; *p* < .001) at Week 2, −0.60 (−0.98 to −0.22; *p* = .002) at Week 3, and −0.52 (−0.91 to −0.13; *p* = .009) at Week 4.

**FIGURE 4:**
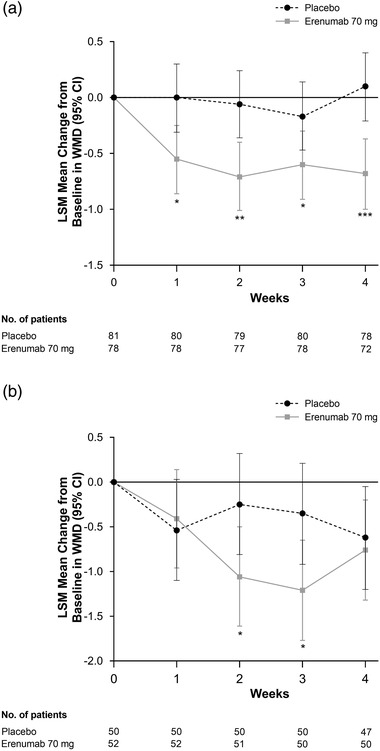
Change from baseline in weekly migraine days at Weeks 1–4 in patients with (a) episodic migraine (EM) and (b) chronic migraine (CM) in Study 20170609. LSM, least squares mean. *p < .05; **p < .01; ***p < .001.

When examining onset of effect by migraine subtype in Study 20170609, a similar pattern of effect was observed, with reductions in WMD observed by Week 1 in both EM and CM subtypes, which were sustained to Week 4 (Figure 4). In patients with EM, LSM changes from baseline (95% CI) at Weeks 1 and 4 were 0.0 (−0.31 to 0.30) and 0.10 (−0.21 to 0.40) for placebo, respectively, versus −0.55 (−0.86 to −0.25) and −0.68 (−1.00 to −0.37) for erenumab 70 mg, respectively. In patients with CM, LSM changes from baseline (95% CI) at Weeks 1 and 4 were −0.54 (−1.10 to 0.03) and −0.62 (−1.20 to −0.05) for placebo, respectively, versus −0.41 (−0.96 to 0.14) and −0.76 (−1.32 to −0.20) for erenumab 70 mg, respectively. In patients with EM, placebo‐adjusted differences in LSM (95% CI) change from baseline in WMD for erenumab 70 mg were −0.55 (−0.97 to −0.12; *p* = .012) at Week 1 and −0.78 (−1.21 to −0.35; *p* < .001) at Week 4. Placebo‐adjusted differences in WMD in favor of erenumab were also observed after Week 2 in patients with CM. Placebo‐adjusted differences in LSM (95% CI) change from baseline in WMD for erenumab 70 mg were 0.12 (−0.59 to 0.84; *p* = .73) at Week 1, −0.81 (−1.53 to −0.09; *p* = .028) at Week 2, −0.86 (−1.58 to −0.14; *p* = .020) at Week 3, and −0.13 (−0.86 to 0.60; *p* = .72) at Week 4. However, effects in this subgroup were confounded by the large placebo effect and small sample size.

## 4 DISCUSSION

In this post hoc analysis of two similarly designed, randomized, double‐blind, placebo‐controlled studies, erenumab treatment was associated with early reductions in WMD compared with placebo. Improvements occurred as early as Week 1 in favor of erenumab and reached significance by Weeks 1─2 in both studies. These improvements were sustained for the duration of the 4‐week evaluation period, with the results of the primary studies confirming that these improvements continued throughout the duration of the double‐blind phases of both studies (Sakai et al., 2019; Takeshima et al., 2021). In both studies, between‐treatment differences in favor of erenumab versus placebo were observed at all time points in the overall population. These differences were significant at all time points for both the 70 and 140 mg erenumab groups in Study 20120309, and at Weeks 2─4 in the overall population in Study 20170609.

When examining onset of efficacy by migraine subtype in Study 20170609, significant reductions in WMD were observed by Week 1 for the EM subtype and Week 2 for the CM subtype, with improvements sustained for the duration of the 4‐week evaluation period. However, results for the CM subtype were confounded by the large placebo effect and small sample size. These results are consistent with the results reported from the post hoc analysis of the global studies (N = 1622) (Schwedt et al., 2018) as well as the known pharmacokinetic profile of erenumab following SC injection (Vu et al., 2017). In the post hoc analysis of the global studies, erenumab treatment was associated with significant reductions in WMD compared with placebo as early as Week 1 for some doses and by Week 4 for all doses (Schwedt et al., 2018). The findings reported in the current analysis are also consistent with results for other anti–CGRP monoclonal antibodies, including fremanezumab (Winner et al., 2019), galcanezumab (Detke et al., 2020), and eptinezumab (Silberstein et al., 2020).

Most of the commonly prescribed oral preventive medications (including beta‐blockers, tricyclic antidepressants, and topiramate) require a prolonged dose titration period of up to several weeks in order to minimize adverse events (Parsekyan, 2000; Topamax [prescribing information], 2009), and even then efficacy may be suboptimal (Kawata et al., 2021; Parsekyan, 2000). Based on their delayed onset of efficacy, the International Headache Society guidelines for controlled trials of preventive treatment recommend a minimum treatment period of at least 12 weeks before assessment of efficacy is performed (Tassorelli et al., 2018). However, studies show that patients in Japan frequently discontinue migraine preventive treatment within the first to 2─6 months of treatment (Kawata et al., 2021; Meyers et al., 2019), with avoidance of side effects and perceived lack of efficacy identified as main reasons patients discontinue migraine preventive treatment (Kawata et al., 2021; Meyers et al., 2019). This suggests that a narrow therapeutic window may exist in which patients make important decisions regarding their adherence to, and persistence with, migraine preventive treatment. The findings from the current analysis and those of others demonstrate the rapid and sustained efficacy of erenumab for prevention of migraine in EM and CM, with onset of efficacy observed as early as Week 1 (Schwedt et al., 2018). Coupled with the favorable safety profile demonstrated across both Japanese and global phase 2 and 3 trials (Aimovig [prescribing information], 2018; Dodick et al., 2018; Goadsby et al., 2017; Tepper et al., 2017), it is anticipated that these therapeutic attributes could contribute to improved patient adherence and outcomes. Further, potential improvements in patient adherence to preventive treatment may also translate into a reduced need for acute migraine medications for the treatment of EM or CM, which are associated with medication overuse headache (Torres‐Ferrús et al., 2020), transformation to chronic migraine (Torres‐Ferrús et al., 2020), and development of adverse events, particularly with frequent, long‐term use (Ong & De Felice, 2018).

Results of the current post hoc analysis are strengthened by the similar designs of the 20120309 and 20170609 studies (prospective, randomized, 24‐week double‐blind, placebo‐controlled, treatment period) and use of erenumab across both EM and CM migraine subtypes. Limitations are inherent to subgroup post hoc analyses, and include the relatively small sample size of some groups, the lack of prespecified hypothesis, and lack of control for *p* value inflation due to multiplicity testing. In this context, all *p* values are nominal and should be interpreted with caution.

Nevertheless, erenumab treatment was associated with significant reductions in WMD compared with placebo, with onset of erenumab efficacy occurring as early as Week 1 in patients with both EM and CM. These improvements were sustained to Week 4 in the current analysis, and continued for the duration of the 24‐week double‐blind phase, as reported previously (Sakai et al., 2019; Takeshima et al., 2021). In addition to the favorable benefit‐risk profile of erenumab (Aimovig [prescribing information], 2018), its early onset of efficacy may prove an important benefit to patients with respect to adherence and contribute to improvements in patient outcomes.

## AUTHORSHIP

All named authors meet the International Committee of Medical Journal Editors (ICMJE) criteria for authorship for this article, take responsibility for the integrity of the work as a whole, and have given their approval for this version to be published.

## CONFLICT OF INTEREST

Koichi Hirata: No funding was received for the current manuscript. Health, Labour and Welfare Sciences Research Grants received by the Ministry of Health, Labour and Welfare, Japan for the current work. Consulting fees received from Lundbeck, Otsuka, Eli Lilly, and Amgen. Payment or honoraria (lectures, presentations, speaker's bureaus, etc.) received from Eli Lilly, Lundbeck, Otsuka, Eisai, Amgen, Pfizer, and Daiichi Sankyo. Takao Takeshima: Payment or honoraria (lecture fees) received from Eisai and Eli Lilly. Fumihiko Sakai: Consulting fees received from Amgen, Eli Lilly, Otsuka, and Lundbeck. Noboru Imai: Payment or honoraria (speaker's bureaus) received from Amgen, Daiichi Sankyo, Eli Lilly, and Otsuka. Yasuhiko Matsumori: Payment or honoraria (lecturer fees) received from Eli Lilly, Otsuka, and Daiichi Sankyo. Yoshihisa Tatsuoka: Advisory fees received from Eli Lilly, Otsuka, and Daiichi Sankyo. Payment or honoraria (lecturer fees) received from Eisai, Otsuka, Kyowa Kirin, Takeda, and Daiichi Sankyo. Grants received for commissioned/joint research received from Amgen, Allergan, Eli Lilly, Otsuka, Kyowa Kirin, and Ono. Yotaro Numachi: Employee of Amgen K.K. and shareholder of Amgen Inc. Ryuji Yoshida: Employee of Amgen K.K. Cheng Peng: Employee and shareholder of Amgen Inc. Daniel Mikol: Former employee of Amgen and held Amgen stock. Current employee of NervGen and hold NervGen stock. Gabriel Paiva da Silva Lima and Sunfa Cheng: Employee and shareholder of Amgen Inc.

## AUTHOR CONTRIBUTIONS

Study design: Yotaro Numachi, Ryuji Yoshida, Cheng Peng, Daniel D. Mikol, Gabriel Paiva da Silva Lima, and Sunfa Cheng. Data acquisition: Koichi Hirata, Takao Takeshima, Fumihiko Sakai, Noboru Imai, Yasuhiko Matsumori and Yoshihisa Tatsuoka. Analyses (including statistical): Cheng Peng. Interpretation of data, manuscript writing, feedback, and revisions: all.

### PEER REVIEW

The peer review history for this article is available at https://publons.com/publon/10.1002/brb3.2526

## Data Availability

Qualified researchers may request data from Amgen clinical studies. Complete details are available at the following: https://wwwext.amgen.com/science/clinical‐trials/clinical‐data‐transparency‐practices/.
